# Negative Effects of Annealed Seed Layer on the Performance of ZnO-Nanorods Based Nitric Oxide Gas Sensor

**DOI:** 10.3390/s22010390

**Published:** 2022-01-05

**Authors:** Pragya Singh, Firman Mangasa Simanjuntak, Li-Lun Hu, Tseung-Yuen Tseng, Hsiao-Wen Zan, Jinn P. Chu

**Affiliations:** 1 Department of Materials Science and Engineering, National Taiwan University of Science and Technology, Taipei 10607, Taiwan; pragyasingh@mail.ntust.edu.tw (P.S.); jpchu@mail.ntust.edu.tw (J.P.C.); 2 Institute of Electronics, National Yang Ming Chiao Tung University, Hsinchu 30010, Taiwan; 3 Department of Photonics and Institute of Electro-Optical Engineering, National Yang Ming Chiao Tung University, Hsinchu 30010, Taiwan; popo2208@gmail.com (L.-L.H.); hsiaowen@nycu.edu.tw (H.-W.Z.); 4 Applied Research Center for Thin-Film Metallic Glass, National Taiwan University of Science and Technology, Taipei 10607, Taiwan

**Keywords:** annealing, ZnO, nanorods, thin-film, gas sensor, metal-oxide-semiconductor, nitric oxide

## Abstract

Nitric oxide (NO) is a toxic gas, which is dangerous for human health and causes many respiratory infections, poisoning, and lung damage. In this work, we have successfully grown ZnO nanorod film on annealed ZnO seed layer in different ambient temperatures, and the morphology of the nanorods sensing layer that affects the gas sensing response to nitric oxide (NO) gas were investigated. To acknowledge the effect of annealing treatment, the devices were fabricated with annealed seed layers in air and argon ambient at 300 °C and 500 °C for 1 h. To simulate a vertical device structure, a silver nanowire electrode covered in ZnO nanorod film was placed onto the hydrothermal grown ZnO nanorod film. We found that annealing treatment changes the seed layer’s grain size and defect concentration and is responsible for this phenomenon. The I–V and gas sensing characteristics were dependent on the oxygen defects concentration and porosity of nanorods to react with the target gas. The resulting as-deposited ZnO seed layer shows better sensing response than that annealed in an air and argon environment due to the nanorod morphology and variation in oxygen defect concentration. At room temperature, the devices show good sensing response to NO concentration of 10 ppb and up to 100 ppb. Shortly, these results can be beneficial in the NO breath detection for patients with chronic inflammatory airway disease, such as asthma.

## 1. Introduction

Air pollution is one of the major worldwide health issues [1]. According to the EU Environmental Protection Agency, 467,000 deaths were recorded in the EU due to high air pollution in 2013 where 40,000 people died in the UK alone because of the bad air quality [2]. The primary air pollutants are toxic gases, and nitric oxide (NO) is one of the most toxic gases that poses a threat to human health [3,4]. Environmental monitoring of these toxic gases and chemicals in the range from 1 ppm to a few ppb by highly sensitive sensors is immensely important to controlling the level of pollutants and avoiding human health risks.

The fundamental prerequisite for fabricating reliable gas sensors is to employ a large surface area sensing layer that helps to develop more active sites on the surface; thus, a higher defect concentration is advantageous. The benefit of developing a metal-oxide-based gas sensor is that it can form a variety of nanostructures, resulting in a larger surface area. As needed, these nanostructures can be adjusted in terms of materials, size, and density. In comparison to 2D (graphene) and polymer materials, metal-oxide-based devices have shown more flexibility and efficiency [5,6]. Moreover, metal-oxide materials are more appealing due to their beneficial features—such as high sensitivity, low cost, simple sensing principle, target gas selectivity, and thermal stability [7,8]. Among them, zinc oxide (ZnO) is one of the most widely used and investigated oxide gas-sensing materials because of its supreme properties such as wide band-gap of 3.37 eV, high melting point (1975 °C), good optical transparency in the visible wavelength region, large exciton binding energy (60 meV) at room temperature, availability in the market, as well as having no toxic effect on the environment. ZnO materials have high mobility of electrons in the material, and good chemical and thermal stability under various operating conditions [9,10]. On the other hand, 1D ZnO nanostructures—such as nanorods, nanowires, nano-combs, nanotubes, and nanobelts—have a potential material structure to improve the gas sensing performance. ZnO nanorods have a high surface-to-volume ratio that enables the exchange of charge carriers during oxidation/reduction mechanism and fast electron transport along the nanorods that enhance the charge concentration [11,12,13,14,15,16].

Various methods have been developed to synthesize ZnO nanorods, such as chemical and physical depositions, spray, and spin coating [17,18,19]. In this work, we used a hydrothermal method due to its simplicity, cost-efficiency, and environmental friendliness [20]. Nevertheless, the properties of the grown ZnO nanorods are determined by the quality of the seed layer. Several efforts have been conducted to improve the quality of the seed layer by annealing. In 2011, P. Suresh Kumar et al. reported that a ZnO seed layer annealed at various temperatures could modify the ZnO nanocrystals [21]. Similarly, Ana Pimentel et al. [22], reported that the high-temperature annealing affects the crystal grains of the seed layer, and increases the reaction ability of the surface of the seed layer. However, the relationship between the properties of the seed layer and sensitivity of gas sensor devices is still less understood. Henceforth, this study examines the impact of annealing of the seed layer on the properties of the grown nanorods and, consequently, how they play a role in the performance of the NO sensor device.

## 2. Experimental Process

### 2.1. Sensor Device Fabrication

**Figure 1:**
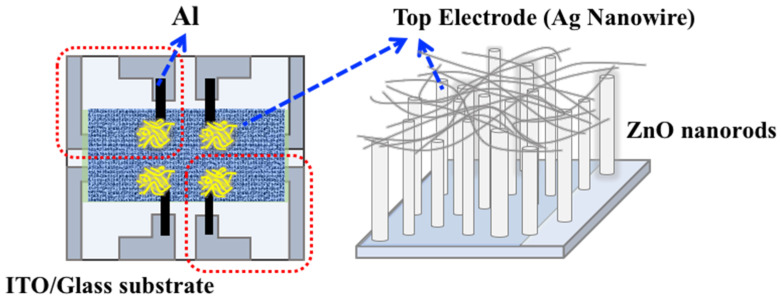
Schematic representation of the fabricated device.

At first, an indium–tin–oxide (ITO)-coated glass with the patterned substrate was cleaned by using acetone and DI water separately for 10 min. Then, thin ZnO seed layers were deposited on the ITO-substrates by using radio-frequency (RF) magnetron sputtering at room temperature in Ar–O_2_ gas ambient. The ZnO seed layer was annealed in air (21% O_2_ + 79% N_2_) and argon ambient for 1 h at 300 °C and 500 °C. Then, the free-standing ZnO nanorods were grown on the ZnO seed layer by using a precursor aqueous solution of ammonium hydroxide (NH_3_OH) and zinc chloride (ZnCl_2_), and to carry out the hydrothermal process, the solution temperature was maintained at 95 °C for 10 min. The hydrothermal growth procedure was adopted and explained in our previously reported studies [23,24]. As a top electrode, silver (Ag) nanowires were placed on top of the selective areas of the ZnO nanorods. An auxiliary electrode of Al was deposited by using a thermal evaporator to connect the silver nanowires as shown in Figure 1.

### 2.2. Response Measurement Set-Up

For the measurement set-up, to inject the target gas with high purity 99.999%, an electrical syringe pump is used to inject into a tube to mix gas with the background dry air gas. This gas mixture was passed through the microfluid system and dry air gas was flowed into the detection chamber. To prevent the fluctuation due to relative humidity, the system was controlled in a low-humidity environment with a fixed relative humidity of 10%. The electrical measurement (I–V) was characterized by using (Keithley Source Meter-model 2400) system. All the details of the measurement system were mentioned and reported in a previous study [10].

### 2.3. Material Characterization

The scanning electron SEM (SU8010-Hitachi) was used to analyze the morphology of ZnO nanorod thin film. To check the crystal structure of ZnO nanorods and seed layer X-ray diffractometry (XRD; D1. Bede Plc.) was employed. To check defects concentrations of nanorod thin film, the X-ray photoelectron spectroscopy (XPS; PHI Quantera SXM) was used and C1s Peak was taken as a reference with a binding energy of 284.5 eV.

## 3. Results and Discussion

**Figure 2:**
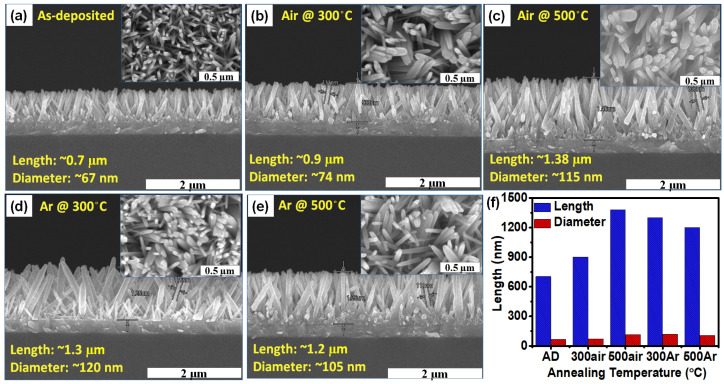
Cross-section SEM images of the grown nanorod film (**a**) as-deposited ZnO seed layer (**b**) annealed in air at 300 °C (**c**) annealed in air at 500 °C (**d**) annealed in argon at 300 °C (**e**) annealed in argon 500 °C (inset shows the top view of the device) and (**f**) shows the comparison results between length and diameter of nanorod thin film.

To observe the changes in the morphology of the ZnO nanorods, we performed a FESEM analysis. Figure 2a–f shows a cross-sectional view of the SEM images of as-deposited and the annealed ZnO seed layer at different ambient at 300 °C and 500 °C for one hour and a comparison of the length and diameter of the nanorods. In contrast, inset images show the top-view SEM images of as-deposited and annealed seed layers, respectively. All the grown nanorod films are perfectly aligned in the vertical direction. SEM images show that annealing seed layer at different temperatures can successfully grow the complete structure of the ZnO nanorods with a denser structure as depicted in the inset images of Figure 2a–e. For the nanorods grown on the annealed seed layer, the length and diameter of the nanorods are longer and thicker than those without annealing, regardless of the annealing temperature. The length of as-deposited seed layer grown nanorod film is about 700 nm, and after annealing the seed layer, the length of the nanorod film increased from 900 nm to 1380 nm as shown in Figure 2b–e. The ZnO nanorods grown at annealed at 300 °C exhibited a morphology close to that of the as-deposited seed grown ZnO nanorods. However, as shown in Figure 2c–e, the tips of the ZnO nanorods gradually became bundles, and the diameter of the nanorods increased with the annealing temperature.

ZnO seed layer plays a significant role in morphology changes of the nanorod thin film [16]. It is mentioned in the literature that when the seed layer is subjected to high-temperature annealing, it will affect the crystal grains in the seed layer, increase the reaction ability of the surface of the seed layer, and then affect the structure of the nanorods [22]. The change in length and diameter of nanorods ultimately depends on the annealing temperature of the seed layer [23]. Due to annealing treatment, the grain of ZnO seed layer aggregated or melting (high temperature). Annealing temperature influences the grain size of ZnO seed layer by aggregated/melting and regrowth under sufficient thermal energy [13,25]. The diameters of the ZnO nanorods were fully determined by the seed layer grain size. The reactivity of the seed-layer surface may be improved by annealing it, which leads to a stronger electrostatic contact between the surface and the ZnO nuclei in the solution [26]. When the seed layer is annealed at a high temperature, thermal energy is excessively high, and this process forces oxygen atoms to escape from the ZnO lattice, leading to the formation of oxygen defects. Surface defects are considered appropriate sites for the interaction of target gas molecules, resulting in an increase in ZnO resistance with electron capture. ZnO nanorods are grown at high temperature annealed seed layer which aggregate with each other can be observed in Figure 2c,e. Overall, the diameter of the zinc oxide nanorods grown by the hydrothermal method increases as the temperature of the seed layer annealing increases as depicted in Figure 2f.

**Figure 3:**
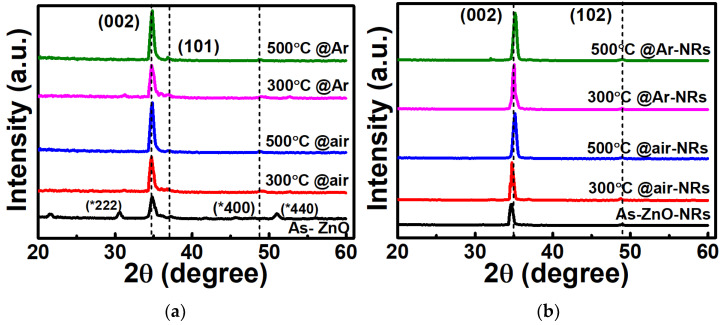
XRD pattern of (**a**) ZnO thin film (as-deposited) and annealed ZnO thin film at 300 °C, 500 °C in dry air, Ar ambient, and (**b**) grown nanorod film on as-deposited ZnO thin film and annealed ZnO film at 300 °C, 500 °C in dry air, Ar ambient; respectively.

Figure 3a,b represents the XRD patterns of the as-deposited ZnO seed layer and annealed ZnO seed layer at 300 °C and 500 °C in air and Ar ambient grew nanorods thin-film, respectively. The crystal structure of the ZnO seed layer and grown nanorod film shows a hexagonal wurtzite structure and matched a preferred orientation of (002) in conformation with the database (JCPDS, no. 36-1451). The peak intensity of the (002) plane is higher than other peaks due to lower surface energy along the (002) direction, so the nanorods grow faster with c-axis orientation. As shown in Figure 3b, the XRD pattern of nanorod thin film, the peak intensity of (002) plane is similar in all the nanorod thin film. The peaks (*222), (*400), and (*440) are from the ITO bottom electrode. These peaks are only observed in ZnO thin films. After growing nanorod film, only a (002) peak with high intensity is observed. All the nanorods are perfectly aligned vertically, as mentioned in cross-section images Figure 2a–e. 

**Figure 4:**
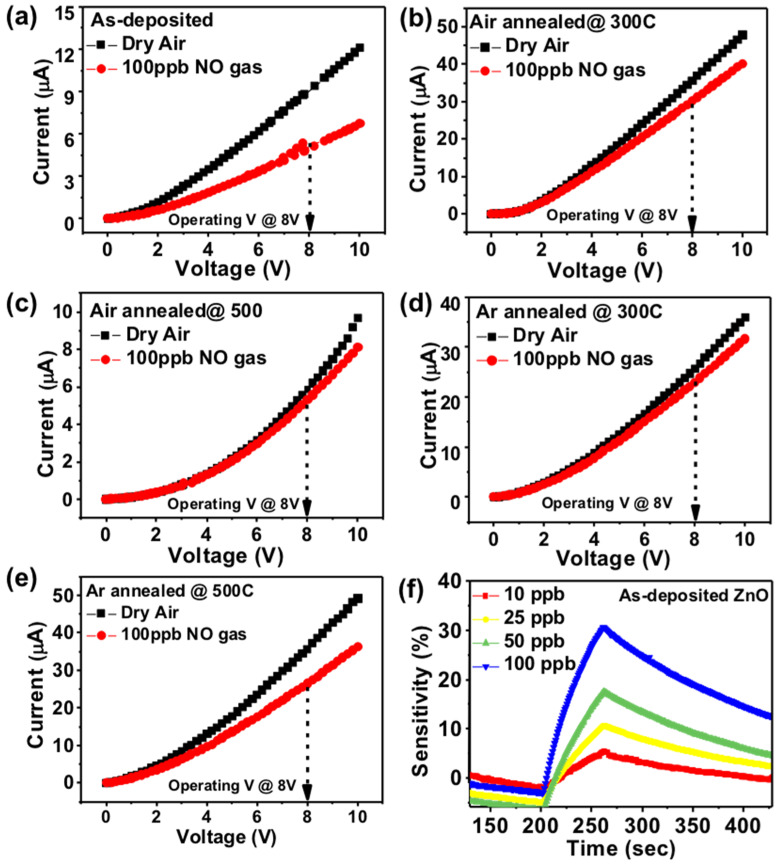
I–V analysis of (**a**) as-deposited (**b**) annealed in air at 300 °C (**c**) annealed in air at 500 °C (**d**) annealed in Ar at 300 °C (**e**) annealed in Ar at 500 °C for 1 h and (**f**) shows the sensitivity (%) of the as-deposited ZnO seed layer grown nanorods to various NO concentrations.

To measure the nitric oxide gas sensing response of the devices, the dry air was used as the background gas and fixed at a flow rate of 750 cc/min, and the voltage and current (I–V) curves of the devices were measured in the dry air and 100 ppb of nitric oxide, as shown in Figure 4a–e. To perform the gas sensing measurements of nitric oxide, a +8 V was applied to the device at room temperature. The I–V results show that the proposed silver nanowires as a top electrode show good conductivity through the ZnO nanorod film towards the ITO bottom electrode. To observe the influence of NO gas on I–V curves, we expose the sensors to 100 ppb NO gas for 30 s and measure the I–V curve again. The current drop is observed after introducing the NO gas to the chamber in all the sensing devices as the results are plotted into Figure 4a–e. The higher current drop of about 45% at 8 V working voltage is observed in as-deposited grown nanorod film due to higher porosity and well-aligned nanorods morphology as depicted in Figure 2a and Figure 3b. In the case of annealing seed layer grown nanorods shows less porous morphology of nanorod thin film, resulting in lower current drop after exposing the sensing layer to NO gas as shown in Figure 4b–e. The current drop in the air annealed seed layer is about 15% (300 °C-Figure 2b) and only 9% (500 °C-Figure 2c) at 8 V. After exposing to NO gas, the current drops by 11% (300 °C-Figure 2d) and 25% (500 °C-Figure 2e) in the case of an argon annealed seed layer-based device. In denser morphology, the exposed area of the sensing layer is reduced; hence, the NO molecule absorption is reduced, resulting in a lower current drop. The sensitivity findings of as-deposited ZnO seed layer nanorod film at various NO gas concentrations are shown in Figure 4f, and we observed that as the NO gas concentration was increased, the sensitivity increased linearly. Here, sensitivity refers to the change of measured signal in accordance with the analyte concentration of the target gas.

To quantify the magnitude of the change, we choose the response value (response) used in most of the literature to define this change [11]. The response value is actually the percentage of the current rate of change, which is defined as

$$\mathrm{Response}=\frac{I_{\left(t=x\right)}-I_{\left(t=0\right)}}{I_{\left(t=0\right)}}\times 100\%$$

where *I*_((*t*=0))_ is the initial current value when the target gas introduced to the sensing layer, and *I*_((*t*=*x*))_ is the current value when the target gas stops passing into the sensing layer, where x is the target gas exposer/sensing time.

**Figure 5:**
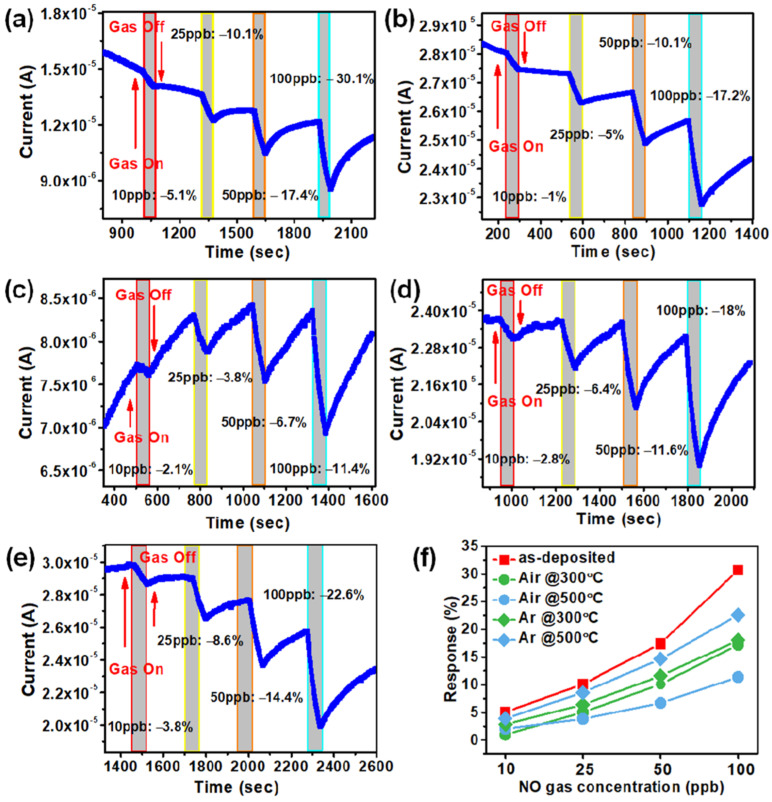
Shows the sensing response and time (s) results (**a**) as-deposited, (**b**) annealed in air at 300 °C, (**c**) annealed in air at 500 °C, (**d**) annealed in Ar at 300 °C, and (**e**) annealed in Ar at 500 °C. (**f**) Summarized gas sensing response of all the devices at 10, 25, 50, and 100 ppb measured at room temperature.

The NO target gas concentration was used at 10 ppb, 25 ppb, 50 ppb, and 100 ppb to check the current change at various concentrations. The observed gas sensing response (%) is mentioned in Figure 5a–e. The highest gas sensing response is observed as-deposited seed layer grown nanorods layer device. At 10 ppb, 25 ppb, 50 ppb, and 100 pb the sensing response is 5.1%, 10.1%, 17.4%, and 30.1%, respectively. The experimental procedure mentioned in the above section, the application of 10 ppb, 25 ppb, 50 ppb, and 100 ppb concentration of nitric oxide, and observe the gas response value, the experimental results are shown in Figure 5. Response is determined by the difference of the current change during gas on and off states.

The result of the reaction of each nanorod with nitric oxide gas is optimal in as-deposited seed layer device. As a result of the actual reaction with nitric oxide gas, the comparison of the components in the seed layer without annealing and different annealing temperatures are shown in Figure 5a–e. It can be clearly seen from these results that the ZnO nanorods sensing layer with as-deposited seed layer is better than the annealed seed layer. The possible reason is that after the annealing of the seed layer, the diameter of the grown zinc oxide nanorods is increased, so a lower porosity introduced and less nanorod area is exposed to the targeted gas. This tradition was followed by all the annealing nanorod thin films. As mentioned in Figure 2a–e, the number of nanorods has significantly increased; however, in the results, gas sensing response decreased.

**Figure 6:**
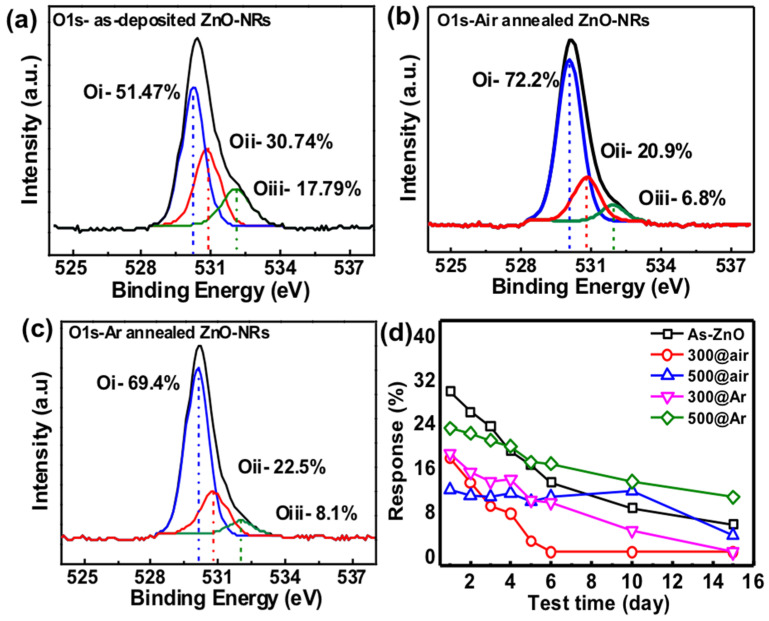
XPS spectra of ZnO nanorod film O1s core level (**a**) as-deposited ZnO seed layer, (**b**) annealed seed layer in air 300 °C, (**c**) annealed seed layer in Ar 300 °C, and (**d**) stability test of all the devices at 100 ppb of NO gas.

To confirm the effect of oxygen defects on gas sensing, we performed the X-ray photoelectron spectroscopy (XPS) analysis of as-deposited and annealed seed layers at 300 °C and 500 °C in ambient argon. The results of the O1s orbital study are shown in Figure 6. Some published literature mentioned that zinc oxide, the oxygen element in the O1s orbital domain, can be dissolved into three peaks [27,28]. Waveforms of different bond energies are superimposed. The three peaks have bond energies of about 530 eV, 531 eV, and 532 eV, and the bond they represent is a metal bond between Zn-O, oxygen vacancies, and others such as OH or CO_3_. From the results of XPS analysis, it can be found that in the O1s orbital domain of each component, the ratio of oxygen vacancy bonding in the annealed device is about 10% lower than that of the as-deposited device, which may also cause the sensing effect after annealing the seed layer. Stability is an important factor of gas sensing devices. To perform the stability test, the devices were measured at 100 ppb NO gas under the same test conditions as before for up to 6 days, 10 days, and 15 days, the observed responses are 29.9%, 27.6%, 21.8%, 23.2%, 25.2%, 20.6%, 10.4% (10th day) and 7.8% (15th day) in as-deposited ZnO seed layer device as shown in Figure 6d, indicating a downward trend in gas response. After annealing the seed layer at 500 °C in ambient argon, it shows good stability up to 6 days, 10 days, and 15 days with a very small variation in gas sensing response as depicted in Figure 6d. The improvement in stability due to annealing treatment is due to its morphology that helps to maintain the gas sensing response; however, in as-deposited seed layer device—due to higher porosity—all the nanorods were exposed to NO gas at once and react. The gas sensor response decreased a few percent due to less recombination site availability [11]. An additional thin film that covers the nanorod thin film to prevent oxidation may help to improve the device’s stability. It is a critical characteristic that will be investigated further in our future research.

### Gas Sensing Mechanism

Metal oxide semiconductors-based gas sensing mechanisms and phenomena have been widely discussed. However, there are still some possible and widely accepted interpretations [29,30,31]. During the gas sensing process, the oxygen vacancy on the surface of the material has a greater activity, the gas molecules are attached to the vacant oxygen position and its change influence the electrical analysis [32]. When the target gas interacts with the sensing layer material, physical reactions occur and this changes physical properties, such as electrical conductivity and resistance, by absorbing the oxygen ions from the sensing material. This physical change functions as the detection (response) of the target gas. In this case with nitric oxide gas sensors, dry air was used as a background gas while exposing the ZnO nanorods sensing layer to the target nitric oxide gas. When NO gas molecules reached the wall of nanorods thin layer, the O-ions adsorbed directly at the surface of the nanorods and NO-ions took the electrons from the nanorod film and this electron reached the conduction band. As a result, the potential barrier between the wall boundaries of nanorods increases due to the change in the charge carrier concentration [33]. This change affects the conductivity of the nanorod thin film [34,35,36]. Then, the change in the concentration of electrons on the surface of the ZnO-NR film decreased and vice versa, the resistance of nanorods increased.

Therefore, we applied a fixed voltage to observe the gas response of nanorod devices in nitric oxide, and used the current changes to determine the response to the gas effect. The oxidation-reduction reaction of nitric oxide on the surface of the metal oxide is shown in the following equation [8].

$$\mathrm{NO}/{\mathrm{NO}}_{2}{}_{\left(\mathrm{gas}\right)}+e^{-}\to {\mathrm{NO}}^{-}/{\mathrm{NO}}_{2}{{}^{-}}_{\left(\mathrm{ads}\right)}$$

As a result, this gas sensing mechanism depends on defects concentration and surface exposure to the target gas [37]. All the devices show sensing response towards NO gas due to ZnO nanorod thin film, and the variation in response is completely influenced by defects availability on the nanorod walls. If the nanorod film is denser, there is less mobility of gas molecules; hence the response decreases. As mentioned in the above results, in Figure 5a, the best sensing response occurs due to higher porosity and oxygen defect concentration, which provides a greater reaction and combination on the nanorods layer. We reported this work due to its contrary annealing effect on gas sensing performance. The annealed seed layer at 500 °C in Ar stability shows better stability than the as-deposited seed layer device. In this work, we employed two annealing conditions as mentioned in the literature; however, in our work, an annealing treatment is not adequate for nitric oxide gas sensing performance due to a change in oxygen defect concentration and morphology.

C. Samanta et al. managed to reduce the working temperature of the sensors to room temperature, but with the high NO gas concentration of 10 ppm the response was 35% [38]. Many studies have shown sensing response at room temperature with ppm level concentrations of NO gas as mentioned in Table 1. Furthermore, P. Luo et al. obtained a 100 ppb NO gas response at 150 °C using nano-spiral ZnO films prepared by glancing angle deposition (GLAD) method [39]. Additional attempts have been made by noble metal doping processes to improve the response while simultaneously lowering the operating temperature. Y.T. Tsai et al. managed to lower the detection limit to 36 ppb NO at 200 °C using an Ag-doped ZnO-based sensing layer [40]. To improve the NO gas sensing sensitivity, either a high temperature or doping the sensing layer with noble metal was required. However, doping with pricey noble metals significantly raises the sensor’s cost. As a result, additional research into new sensing materials with ppb-level sensitivity, great selectivity, and low cost is desirable.

## 4. Conclusions

In conclusion, we investigated the influence of the annealing seed layer on nanorod thin films and gas sensing response. The sensor exhibits ZnO nanorods grown by hydrothermal method and fabricated in a vertical device structure. The ZnO nanorod film grown on an as-deposited ZnO seed layer was found to be more sensitive towards nitric oxide gas. At room temperature (~26 °C), the ZnO-nanorod sensor was found to be very sensitive towards nitric oxide gas. The gas sensing response was measured at 10 ppb to 100 ppb of NO gas with response rates of 2.1% to 30.0%, respectively, in a dry air background. Finally, by comparing sensor performance of nanorods grown on different annealed seed layers, we propose that gas sensing response is mostly influenced by the oxygen defect concentration and microstructures of the nanorods. Although all of the devices demonstrated gas sensing responses at room temperature, after annealing treatment in air and ambient argon, the defect concentration was reduced, and the nanorod morphology became denser; thus, the sensing response was reduced. Our future research will address and resolve the issue of long-term stability.

## Figures and Tables

**Table 1:** Comparison for nitric oxide gas-sensing performance of this work with reported ZnO-based sensors.

No.	Sensing Materials	Operating Temp. (°C)	Concentration	Response (%)	References
1.	ZnO nanocrystals/N-rGO	90	100 (ppb)	2	[41]
2.	Ag-doped ZnO	200	36 (ppb)	90	[40]
3.	ZnO/p-Si nanowires	RT	10 (ppm)	35	[38]
4.	ZnO-NRs thin film	RT	1 (ppm)	57.1	[11]
5.	ZnO nanostructure	RT	40 (ppm)	23.6	[42]
6.	ZnO/CdO nanofibers	215	3 (ppm)	1.7	[43]
7.	ZnO nanowire	RT	5 (ppm)	15	[44]
8.	ZnO microtubules	92	10 (ppm)	78.5	[45]
9.	Nanospiral ZnO film	150	100 (ppb)	16.9	[39]
10.	ZnO-NRs (as-deposited)	RT	100 (ppb)	30	This work
